# Variations in self-regulation of behaviour among different groups of the Russian population during the second wave of the COVID-19 pandemic

**DOI:** 10.1192/j.eurpsy.2024.1067

**Published:** 2024-08-27

**Authors:** V. I. Rozhdestvenskiy, V. V. Titova, I. A. Gorkovaya, D. O. Ivanov, Y. S. Aleksandrovich

**Affiliations:** Department of Psychosomatics and Psychotherapy, Saint Petersburg State Pediatric Medical University, Saint Petersburg, Russian Federation

## Abstract

**Introduction:**

During a pandemic, the population is required to adapt effectively to drastically altered environmental conditions to avoid the development of psychiatric disorders or other maladaptive responses. This adaptation is closely linked to an individual’s ability to regulate their behaviour effectively and to develop traits such as pliability and autonomy.

**Objectives:**

The research aims to investigate individual self-regulation among students studying humanities disciplines and individuals living with HIV during the second wave of the COVID-19 pandemic in Russia.

**Methods:**

Data collection took place from January to July 2021 using a custom-designed Google form. The study involved 35 university students in Russia specializing in humanities and 59 individuals living with HIV. To assess the development of individual self-regulation and determine its specific profile, we utilized the “Behavioural Self-Regulation Style” questionnaire developed by V.I. Morosanova.

**Results:**

We found that 43% of students have an average level of self-regulation, 37% - high and 20% - low. Among people living with HIV the distribution is similar: 53 % have an average level of self-regulation, 37 % - high and 10 % - low. The analysis of average results of the scales did not reveal statistically significant differences among the groups of respondents. The average profiles have no pronounced peaks and look as follows: planning (M = 5.77±2.16 - students, M = 6.24±1.90 - patients, p > 0.05), modelling (M = 5.26±1.80 vs M = 5.69±1.90, p > 0.05), programming (M = 6.00±1.50 vs M = 5.93±1.66, p > 0.05), performance evaluation (M = 6.26±1.42 vs M = 5.78±1.60, p > 0.05), pliability (M = 6.17±1.87 vs M = 6.58±1.90, p > 0.05) and autonomy (M = 5.00±2.33 vs M = 5.56±2.08, p > 0.05) were almost at the same level in both the student and patient groups.

**Conclusions:**

During the second wave of the COVID-19 pandemic in Russia, there were no significant distinctions observed in the self-regulation behaviour styles between students and individuals living with HIV. The majority of participants from these chosen groups demonstrated a similar average level of effectiveness in self-regulating their behaviour, as well as comparable degrees of pliability and autonomy development.

**Disclosure of Interest:**

None Declared

